# Relating drug response to epigenetic and genetic markers using a region-based kernel score test

**DOI:** 10.1186/s12919-018-0154-5

**Published:** 2018-09-17

**Authors:** Summaira Yasmeen, Patricia Burger, Stefanie Friedrichs, Sergi Papiol, Heike Bickeböller

**Affiliations:** 1 Department of Genetic Epidemiology, University Medical Center, Georg-August University Göttingen, Humboldtallee 32, 37073 Göttingen, Germany; 2 Department of Psychiatry and Psychotherapy, Ludwig Maximilian University Munich, Nussbaumstrasse 7, 80336 Munich, Germany; 3 Institute of Psychiatric Phenomics and Genomics (IPPG), Medical Center of the University of Munich, Nussbaumstrasse 7, 80336 Munich, Germany

## Abstract

In GAW20, we investigated the association of specific genetic regions of interest (ROIs) with log-transformed triglyceride (TG) levels following lipid-lowering medication using epigenetic and genetic markers. The goal was to incorporate kernels for cytosine-phosphate-guanine (CpG) markers and compare the kernels to a purely parametric model. Post-treatment TG levels were investigated for post-methylation data at CpG sites and region-specific SNPs and adjusted for pre-treatment TG levels and age, in independent individuals only (real data: *n* = 150; simulated data, replicate 84: *n* = 111). In both data sets, our single-CpG-marker results using kernels and linear regression were in good agreement. In the real data, we investigated the introns of the *CPT1A* gene previously reported as associated with TG levels as separate ROIs, and were able to find hints of an association of cg17058475 and cg00574958 with post-treatment TG levels. In the simulated data, we investigated a total of 10 regions, in which the 5 causal and 5 non-causal markers lie, respectively, with increased methylation variances, yielding plausible results for the 3 window sizes. Overall, this indicates that kernels for CpG markers are feasible. An interaction regression model for the causal SNP with the nearest CpG marker identified an effect for the SNPs with the three greatest heritabilities simulated. The simulation model assumed full SNP effect only for unmethylated regions decreasing to zero in the case of full methylation. Thus, in the context of a clear candidate setting, interaction between epigenetic and genetic data may enhance information, albeit nominally, even with small sample sizes. Relieving the burden of multiple testing, developing kernels further to analyze data from multiple omics jointly is well warranted.

## Background

The human genome is a highly intricate system comprising various genic and gene regulatory elements. Epigenetic intervention turns it into a jungle. High-throughput technologies have been used to profile phenotypes in multiple omics dimensions. In order to dissect complex genomic traits, statistical tools need to handle a multitude of markers both within and across such dimensions. The kernel score test (KST) enables us to test a set of markers for an overall association with a phenotype [1], such as those markers within a region of interest (ROI). It highly reduces the burden of multiple testing without simply aggregating the data. KST can be applied to common and rare variants, or adjusted for covariates and applied to data of genome-wide association studies (GWAS), epigenome-wide association studies, or sequence data (where it was named the sequence kernel association test [2]).

Our previous research [3, 4] focused on genetic data and mainly on logistic regression. In the current analysis, we focused on the use of the KST, employing methylation markers to investigate a normally distributed drug response in independent participants. In several regressions we modeled post treatment log-transformed triglyceride (post-lnTG) as a function of epigenetic and/or genetic markers or kernels thereof, adjusting for pre-treatment log-transformed triglyceride (pre-lnTG) and age. We initially investigated the use of KST for epigenetic markers alone and then with genetic markers. We analyzed both the real and simulated data for a selection of candidate ROIs.

## Methods

### Data

The GAW20 data were provided by the Genetics of Lipid Lowering Drugs and Diet Network (GOLDN) study [5], a longitudinal family-based study involving 991 participants of European descent to localize novel loci contributing to triglyceride (TG) response in connection with fat loading and fenofibrate treatment. Data were collected at 4 time points: visits 1, 2, 3, and 4. We performed a pre−/post-treatment analysis using only visits 2 and 4, as the time between visits 1, 2 and 3, 4 was only one 1 day and the pre- and post-methylation data were only recorded at visits 2 and 4. To normalize distributions, TGs were log-transformed (pre-lnTG and post-lnTG). In addition, we used post-methylation data, GWAS data, and age.

GAW20 also provided simulated data using a family structure and genotypes identical to those in the original data. The answer sheet we were provided described the simulation model. Post-treatment methylation levels were modeled based on pre-methylation with a higher variation at ten 10 CpG cytosine-phosphate-guanine (CpG) markers than for all others. TGs were influenced by five 5 causal SNPs with decreasing heritability and several polygenes. However, the influence of each of the 5 causal SNPs on log TG change decreased with increasing degree of methylation in that particular ROI. Five of the 10 CpG markers were those close to the causal SNPs. We defined the corresponding region as causal ROI and the other mentioned regions as non-causal ROIs.

Since we were focusing only on independent individuals with available phenotypic and genetic/epigenetic information, we only included exactly one member from each pedigree. Hence, we included 150 individuals from the real data and 111 individuals from the simulated data of replicate 84.

### Selection of ROIs

We first investigated models with and without kernels with post-treatment methylation data. To define ROIs for the simulated data, we consulted the GAW20 answers for the 5 causal and 5 noncausal CpG markers and associated SNPs to define 10 ROIs with the boundaries lying 0 kilobase pairs (kbp), 15 kbp, and 3 kbp upstream and downstream of each of these 5 + 5 CpG markers. In the real data, we formulated sets of CpG markers in the *CPT1A* gene defined by intronic boundaries. All the genomic information is in build hg18. After this we began to investigate genetic markers and used the knowledge of the model for effect simulation.

### Regression models and KST

To investigate the association of specific ROIs with post-lnTGs, we employed linear and semi-parametric kernel regressions, all adjusted for age and pre-lnTG.

In the KST, we used a linear kernel to transform the available epigenetic (or genetic) information of the n individuals into a similarity matrix *K*. This is calculated as ***K*** = ***ZZ***^***t***^, where ***Z*** is a *n* × *m*_*u*_ matrix for n individuals and *m*_*u*_ markers of region u. It models a linear effect of the considered markers on the response Y. Let *Y = (Y*_*1*_*,…,Y*_*n*_*)* denote the post-lnTGs. *Y* is modeled as:

1 $$ \mathrm{Y}={\mathrm{Xb}}^{\mathrm{T}}+\mathrm{h}\left(\mathrm{Z}\right)+\upvarepsilon $$

where *X* is the matrix for known fixed covariates, including age and pre-lnTG; *b* is the vector of corresponding regression parameters; and *ε* denotes the usual residuals. The non-parametric function *h(Z)* depends on the *n × n* dimensional kernel matrix *K* (for more details refer to Schaid [1]). The KST investigates whether the epigenetic (or genetic) covariance component *h(Z)* equals zero or not. It is computed from maximum likelihood estimates for the parameters of the null model. The *p* values were calculated using Davies’ exact method [6] with the R package CompQuadForm [7].

To investigate CpG markers only, we employed a linear regression that included the marker itself, as well as a kernel regression including a kernel for the ROI. For this kernel, we used three different windows, all of which included the CpG marker itself and windows of sizes ±0 kbp, ±15 kbp, and ± 3 kbp. After investigating CpG markers only, we applied a linear regression including the causal SNP marker ***x***_**1**_ with its corresponding/nearest CpG marker ***x***_**2**_ and their interaction for the 5 causal SNP markers:

2 $$ \mathbf{Y}={\beta}_0+{\beta}_1{x}_1+{\beta}_2{x}_2+{\beta}_{12}\left({x}_1\times {x}_2\right)+\mathrm{covariates}+\upvarepsilon $$

*p* Values are not adjusted for multiple testing. The significance level is set to 5%. All analyses were performed in R.

## Results

### Simulated data

We performed region-based KST and linear regression to analyze the association of the post-treatment methylation in the 10 candidate ROIs with post-lnTG adjusted for pre-lnTG and age. Table 1 lists the *p* values for the kernel with the candidate CpG marker and windows of sizes ±15 kbp, ±3 kbp, and ± 0 kbp, and for single-marker regression analysis. Other than ROI-1, the results for the KST with several CpG markers (±15 kbp, ±3 kbp) are comparable. Furthermore, the results for a single CpG marker (KST ± 0 kbp, simple regression) are also similar. As to be expected from the simulation, no significant associations were found.

**Table 1:** Simulated data: association of 10 candidate CpG markers and their ROIs with post-lnTG adjusted for pre-lnTG and age

ROI	CpG ID	KST, Window Size			Regression
		±15 kbp	±3 kbp	±0 kbp	
ROI-1	cg00000363	0.86	0.15	0.37	0.49
ROI-2	cg10480950	0.09	0.09	0.58	0.63
ROI-3	cg18772399	0.65	0.65	0.56	0.57
ROI-4	cg00045910	0.61	0.71	0.73	0.89
ROI-5	cg01242676	0.49	0.33	0.49	0.57
ROI-6	cg00703276	0.13	0.13	0.53	0.62
ROI-7	cg01971676	0.51	0.51	0.97	0.98
ROI-8	cg11736230	0.79	0.83	0.22	0.18
ROI-9	cg12598270	0.15	0.15	0.69	0.81
ROI-10	cg00001261	0.78	0.79	0.58	0.61

*p* Values were computed by KST with varying window sizes including the CpG marker or by single-marker linear regression

Subsequently, we performed the KST that incorporated genetic information by employing the causal SNPs nearest to the CpG markers considered above. As expected, this did not yield significant associations (data not shown). Lastly, for the 5 causal ROIs, we employed the regression model defined by eq. (2) with causal SNP, nearest CpG marker, and their interaction. Table 2 presents the results. We found nominally significant associations for the first three SNPs in ROI-1, ROI-2, and ROI-3. The SNPs rs9661059, rs736004, and rs1012116 had the greatest heritabilities of the 5 causal SNPs (see GAW20 answers). These effects could only be detected by including the nearest CpG marker. Three significant main effects (for rs9661059, rs736004, and rs1012116) and two significant interaction effects were found (Table 2). Our results are in good coherence with the simulation setup of GAW20, in which the effect of the causal SNP is at a maximum in an unmethylated region and decreases as the degree of methylation in the region increases.

**Table 2:** Simulation data: association of 5 causal SNPs and their nearest CpG marker with post-lnTG, adjusted for pre-lnTG and age

ROI	CpG ID	SNP ID	CpG Marker	SNP	CpG × SNP
ROI-1	cg00000363	rs9661059	0.0846	0.0187	0.0484
ROI-2	cg10480950	rs736004	0.0192	0.0237	0.0192
ROI-3	cg18772399	rs1012116	0.1447	0.0367	0.1933
ROI-4	cg00045910	rs10828412	0.9252	0.4915	0.9708
ROI-5	cg01242676	rs4399565	0.0649	0.3519	0.0756

*p* Values of interaction model Eq. 2

### Real data

We analyzed the introns of the *CPT1A* gene. Table 3 presents all the results. Intron 1 includes the CpG markers previously reported as associated with (baseline) TG [5]. Thus, we investigated single CpG markers of that intron and found two hints for associations with post-lnTG for cg17058475 and cg00574958 (Table 4). Again, the KST and single marker linear regression are in good agreement.

**Table 3:** Real data: association of sets of CpG markers in 14 introns of the *CTP1A* gene with post-lnTG, adjusted for pre-lnTG and age

Intron number(Int)	Int1	Int3	int 4	Int5	Int 6	Int7	Int 9	Int10	Int12	Int13	Int14
*p* Value	0.08	0.74	0.03	0.76	0.95	0.46	0.12	0.09	0.01	0.46	0.59

*p* Values computed by KST

**Table 4:** Real data: association of 4 CpG markers in intron 1 of the *CPT1A* gene with post-lnTG, adjusted for pre-lnTG and age

CpG ID	KST	Regression
cg00574958	0.066	0.070
cg09737197	0.271	0.276
cg17058475	0.047	0.048
cg01082498	0.285	0.290

*p* Values computed by KST and single-marker regression

## Discussion

In this analysis, the small sample size that results from using only independent individuals limits our power. Nevertheless, we were still able to detect nominally significant associations for 3 of the 5 causal SNPs from the simulation employing a model of interaction with their nearest CpG marker. We also found hints for association of cg17058475 and cg00574958 in intron 1 of the *CPT1A* gene with TGs in the real data by employing KST and the linear regression model. The GOLDN study also reports cg17058475 and cg00574958 are also reported by GOLDN study to be as associated with TGs and cg00574958 as correlated with *CPT1A* expression [5]. Working on the simulated data, we investigated CpG markers in 10 ROIs. A region several kbp in size contains far fewer CpG markers than SNP markers. We revealed for the KST that the window size 0 kbp is similar to linear regression and higher window sizes are similar to each other, yet different from 0 kbp. As no direct CpG effect was modeled, no additional conclusions can be drawn. However, the application of the kernel proves feasible with CpG markers, and not only with genetic markers [3, 4]. Here the use of the kernel is not crucial, as the effect was only given by a simulation model for the causal SNP and the nearest CpG marker, not involving other markers in the region.

The most common design for a treatment-response study is a cohort design with independent people that requires individuals to take the treatment. This might often be unethical for families as a whole (albeit reasonable in the GOLDN study). As we wanted to see the behavior of kernels and regression in this general context, we opted to focus on unrelated individuals. Our strategy can be adapted for family data. In GAW19, we used KST on family data by introducing a design matrix—***Zc***^***T***^ in Eq. (1)—where random effects *c* caused by familial polygenic background are adjusted for **Y** and ***Y*** *= Xb*^*T*^ *+ Zc*^*T*^ *h(Z) + ε* (for more details, see Malzahn et al. [3]). Several GAW20 contributions to this volume used the theoretical or the estimated kinship matrix to construct the random effect in a linear mixed model.

## Conclusions

Our analysis with multi-omics data in a linear regression interaction model in comparison to single omics data in KST and linear regression framework emphasizes that careful integration of multi-omics data might enable researchers to explain a greater proportion of the variance in complex traits, even in small samples. Consequently, it would seem highly warranted to extend kernels to incorporate multiple types of omics data.
